# The Role of Language in Shaping Cultural Perceptions Within Healthcare and Supporting Neurodivergent People's Well‐being and Access to Care: Focus on Autistic Experiences

**DOI:** 10.1002/jmrs.70096

**Published:** 2026-05-19

**Authors:** Ben Potts, C. J. Parish, Nmesoma Francisca Ukaji, Georgia Pavlopoulou, Themis Karaminis, Matthew Lane, James McStravick, Madeline O'Brien, Jane Harvey‐Lloyd, Sophie Shephard, Jane Green, Anne Collis, Nikolaos Stogiannos, Emily Skelton, Christina Malamateniou

**Affiliations:** ^1^ CRRAG Research Group, Division of Radiography, Department of Allied Health, School of Health and Medical Sciences City St George's University of London London UK; ^2^ Radiology University Hospital Southampton NHS Foundation Trust Southampton UK; ^3^ Department of Radiotherapy Planning & Dosimetry, Radiotherapy Physics Ipswich Hospital, East Suffolk and North Essex NHS Foundation Trust Ipswich England UK; ^4^ Norfolk and Norwich University Hospital NHS Foundation Trust Norwich England UK; ^5^ Group for Research in Relationships in Neurodiversity (GRRAND), Department of Clinical, Education and Health Psychology, Division of Psychology & Language Sciences, Faculty of Brain Sciences University College London London UK; ^6^ Anna Freud National Centre for Children and Families London UK; ^7^ Department of Psychology and Neuroscience City St George's University of London London England UK; ^8^ Bedfordshire Hospitals NHS Foundation Trust Luton UK; ^9^ Department of Allied Health Professionals, School of Human and Health Sciences University of Huddersfield Huddersfield UK; ^10^ NHS Greater Glasgow and Clyde Board HQ Glasgow UK; ^11^ LICAMM, Faculty of Medicine & Health University of Leeds Leeds UK; ^12^ School of Allied Health, Exercise and Sports Sciences Charles Sturt University Wagga Wagga New South Wales Australia; ^13^ SEDS Connective Neurodivergence Hypermobility Charity (Symptomatic Hypermobility EDS HSD NeuroDivergence) Sussex Sussex UK; ^14^ ACoRNS, [Autistic Community Research Network Southampton] University of Southampton Southampton UK; ^15^ Independent Researcher London UK; ^16^ Magnitiki Tomografia Kerkiras Corfu Greece

## Abstract

Chronic stigmatisation and social exclusion of neurodivergent people have resulted in poorer quality of life and adverse health outcomes. Language has been weaponised and has furthered their suffering and isolation. With this paper, we propose some considerations of optimal communication interactions in healthcare with neurodivergent people, in general, and autistic people, in particular, to ensure respectful and human‐centred patient and practitioner interactions across both clinical and academic practice.

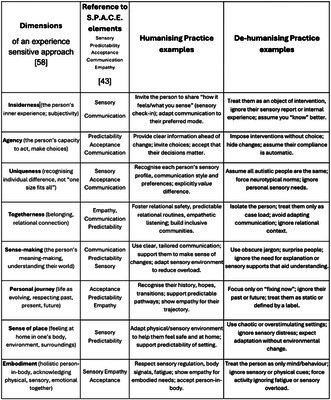

## 1 Introduction

Language is crucial in shaping ideologies, societal perceptions, and attitudes, especially in the context of neurodiversity, the naturally occurring variation in human brain function and cognitive processing, recognising that all brains are unique [1, 2]. This is particularly relevant to all aspects of healthcare, which is truly a tangle of social interactions between practitioners, patients and their families/carers. Within radiography, effective communication is not only the foundation of compassionate, person‐centred practice, but a fundamental component of safety in clinical settings characterised by potent technologies and potential risks. This dynamic requires patients to place considerable trust in practitioners, who are, therefore, encouraged to choose their language carefully, as it carries particular weight [3, 4, 5, 6]. The aim of this editorial is to provide some generic recommendations for optimal use of language when working with neurodivergent patients in general, and autistic people in particular, and it is addressed at MRPs working in education, research, policy‐making and clinical practice, as well as at trainees and students.

## 2 Historical Context

Autism is part of neurodivergence, an umbrella term signifying a different neurocognitive experience from what has been considered ‘typical’. About 1 in 7 people are neurodivergent (and about 1 in 50 are autistic), and this impacts the way they perceive the world, communicate and process environmental stimuli. The history of autism is deeply entwined with medical and psychiatric traditions that framed it as a pathology. The earliest detailed clinical descriptions came from Sukhareva in the 1920s, who described children with rich emotional lives, sensory sensitivities, and distinctive social styles [7, 8, 9]. Her accounts pre‐dated the better‐known works of Leo Kanner, who described ‘early infantile autism’ [10] in 1943, and Hans Asperger, who published on ‘autistic psychopathy’ [11] in 1944. Although written in the wider social context of her time, Sukhareva's tone was strikingly humane, recognising intelligence, conscientiousness, and emotional depth alongside social difference. Her work was largely ignored in the West, likely due to gendered and geopolitical biases [12]. In its place, a pathology narrative dominated in the early 1900s. The ‘refrigerator‐mother’ hypothesis and theory‐of‐mind accounts portrayed autistic people as lacking empathy, reinforced deficit models and entrenched stigma [13, 14].

Recent decades have seen a major epistemic shift. Autistic self‐advocates and scholars have reframed autism within the neurodiversity paradigm, positioning it as a natural form of human variation rather than a disorder [15, 16]. Furthermore, contemporary research increasingly emphasises participatory and community‐based approaches that recognise autistic and neurodivergent people as co‐creators and co‐authors of knowledge, enabling them to reclaim ownership of their narratives [17, 18].

Person‐centred care requires recognition of the whole person, respecting their values, including all aspects of identity, such as neurodivergence, and the creation of environments in which these identities can be expressed safely and authentically [19, 20].

## 3 The Implications of Language and Communication About Autism and Neurodivergence in Healthcare Contexts

In our view, language is closely tied to feeling ‘seen’, and its use can shape neurodivergent people's sense of self and belonging, as well as how they are supported and included in decisions about their care. The inappropriate use of language towards neurodivergent people has promoted a culture of exclusion and stigma, reducing equitable access to housing, education, healthcare, employment, entertainment and minimising the opportunities of neurodivergent people to lead fulfilling lives [3, 21, 22, 23, 24]. This systemic exclusion can create distress and directly impact healthcare outcomes, life expectancy, and mental health amongst the neurodivergent population [23]. The neurodivergent population, where more research exists to demonstrate healthcare inequalities, is predominantly autistic people. Autistic people exhibit a higher prevalence of physical health concerns, such as connective tissue disorders, chronic pain (often accompanied by trauma disbelief and challenges with pain identification), autonomic dysfunction (a disorder causing malfunction of the autonomic nervous system impacting blood pressure, temperature, heart rate and digestion), fatigue, interoceptive or proprioceptive differences (on how they perceive and interpret internal and external bodily signals), alexithymia (the difficulty in experiencing, identifying, and expressing emotions) as well as mental health concerns, such as depression, anxiety, self‐harm and suicidal ideations [23, 25, 26, 27]. Also, their life expectancy is 16–30 years lower than that of their non‐autistic counterparts, depending on co‐existing (or not) learning disability [23, 28]. Deficit‐based language may also discourage them from disclosing their neurodivergence or lead to masking/camouflaging [22, 29, 30, 31, 32]. This limits access to required support, which culminates in non‐attendance for appointments, further impacting their mental health and overall well‐being [21, 22, 31, 33, 34, 35].

In some contexts, autism is described using language drawn from medical models of disease, which emphasise a separation between the individual and the condition, placing priority on the former. This approach has contributed to the use of the so‐called ‘person‐first’ framings, including phrases such as person with autism. By contrast, ‘identity‐first’ expressions—autistic person—reflect the inseparability of autism from self and culture, portraying how being autistic is central to an individual's human experience and how they perceive and interpret the world [14, 18, 36, 37, 38, 39]. Despite recent research and advocacy, language used to describe or address autistic and neurodivergent people may still include medicalised words such as ‘disorder’, ‘abnormality’, ‘profound autism’ or ‘challenging behaviours’, causing further stigmatisation and distress [40, 41]. Functioning labels have also been used to describe neurodivergent people under the assumption that some individuals are more capable of participating in society than others [2]. ‘High functioning’ has been used to describe autistic people without Intellectual Difficulties (ID also called Learning Disabilities) who are therefore assumed to require little to no support to carry out their day‐to‐day activities, whereas ‘low functioning’ has been employed to refer to those with ID and who require consistently high levels of support to undertake daily activities [2]. While terms like low‐ and ‘high‐functioning’, ‘mild’ to ‘severe’ or ‘profound’ autism have been used previously, it is pertinent to note that ‘specific support needs’ is the preferred term within the autistic community [40]. Hence, strong advocacy for person‐centred care in the language used for neurodivergence within healthcare is required.

While these expressions are used without the intention of harm, they promote a culture that dehumanises and misrepresents autistic and neurodivergent people [18, 42], and this may impact their well‐being. Moving away from language that frames conditions primarily as ‘disorders’ or ‘symptoms’ towards terminology that foregrounds the person, their needs, and the dimensions of their lived experience can fundamentally reshape clinical interactions and patient engagement. The words that clinical practitioners, including radiographers, use can redefine quality of care from ‘simple awareness’ of neurodivergence towards ‘intentional affirmation’ [20, 21, 40, 41]. This choice can signal safety, trust and be an act of resistance, countering decades of marginalisation and stigma [19]. Similarly, this paradigm shift can impact practitioners themselves; it encourages them to see beyond pathology, appreciate diversity, foster empathy and understand that language can enhance patient trust and engagement at every stage, from triage and initial assessment to diagnostic procedures, interpretation, and treatment of any clinical condition. It can also create greater psychological safety at work for neurodivergent radiographers.

## 4 Frameworks for Inclusive Healthcare in Clinical Practice

In the sensory‐intensive and often anxiety‐inducing environment of radiography, respectful language mandates specific clinical adjustments to the patient pathway, which are essential for mitigating distress [43, 44]. These adjustments may align with the ‘Autistic SPACE framework’, covering Sensory, Predictability, Acceptance, Communication, and Empathy—a holistic model placing communication at its centre [25]. Communication needs to be framed to overcome the ‘Double Empathy Problem’ [45, 46], recognising that breakdowns are not due to a deficit in the autistic patient, but rather a mutual difficulty in reciprocal communication and understanding [47, 48].

Many autistic people have a much more direct communication style, which may not match with more abstract or vague forms of communication that are more common amongst neurotypical folk. Consequently, radiographers are encouraged to employ literal and explicit language, deliberately circumventing all idioms or vague phrasing. This practice is strongly supported by the ‘Monotropism Theory’ [49], which suggests autistic attention is highly focused. Therefore, providing precise, unambiguous directives prevents the misdirection of attention during a critical procedure [46, 49]. An example for this could be ‘Please stay very still for the next five seconds’, instead of colloquialisms such as ‘Just hang in there’. Furthermore, clinicians need to consider de‐prioritising reliance on non‐verbal cues (tone, expression) as these are prone to misinterpretation [44], opting instead for direct verbal questioning, such as ‘Are you ready to continue?’

Crucially, systemic support necessitates providing alternative communication channels to facilitate process transparency. Systemic barriers, such as difficulty booking by phone or stressful waiting environments, are already recognised as accessibility issues [23, 50, 51]. Offering a written or pictorial guide—frequently termed a ‘social story’—detailing the procedural steps before or upon arrival mitigates the anxiety resultant from uncertainty [52, 53, 54]. This accommodation supports the needs of many autistic people for routine and predictability, which can be disrupted by the unfamiliar context of the diagnostic and/or therapeutic radiography departments. Furthermore, providing accommodations, like online booking, is recommended to maximise predictability [43, 53]. Ultimately, this person‐centred model requires the professional to consult the patient on their preferred use of language and communication style, recognising their unique expertise in their own experience [14, 38, 41, 55, 56, 57].

Several frameworks have been proposed to make healthcare more inclusive, experience‐sensitive and attuned to autistic and neurodivergent people. Table 1 provides a summary organised around key dimensions of an experience‐sensitive approach [58] and the elements of the SPACE framework [25], illustrated with examples of humanising and dehumanising care in healthcare settings.

**TABLE 1:** Recommendations for best practice in healthcare for neurodivergent people, focusing on autistic experiences, building on the dimensions of an experience‐sensitive approach [58] and the SPACE framework elements [25]. Specific examples of humanising and dehumanising practices are also presented here.

Dimensions of an experience‐sensitive approach [58]	Reference to S.P.A.C.E. elements Sensory Predictability Acceptance Communication Empathy [25]	Humanising practice examples	De‐humanising practice examples
**Insiderness** (the person's inner experience; subjectivity)	Sensory Communication	Invite the person to share ‘how it feels/what you sense’ (sensory check‐in); adapt communication to their preferred mode	Treat them as an object of intervention, ignore their sensory report or internal experience; assume you ‘know’ better
**Agency** (the person's capacity to act, make choices)	Predictability Acceptance Communication	Provide clear information ahead of change; invite choices; accept that their decisions matter	Impose interventions without choice; hide changes; assume their compliance is automatic
**Uniqueness** (recognising individual difference, not ‘one size fits all’)	Sensory Acceptance Communication	Recognise each person's sensory profile, communication style and preferences; explicitly *value* difference	Assume all autistic people are the same; force neurotypical norms; ignore personal sensory needs
**Togetherness** (belonging, relational connection)	Empathy, Communication, Predictability	Foster relational safety, predictable relational routines, empathetic listening; build inclusive communities	Isolate the person; treat them only as a case load; avoid adapting communication; ignore relational context
**Sense‐making** (the person's meaning‐making, understanding their world)	Communication Predictability Sensory	Use clear, tailored communication; support them to make sense of changes; adapt the sensory environment to reduce overload	Use obscure jargon; surprise people; ignore the need for explanation or sensory supports that aid understanding
**Personal journey** (life as evolving, respecting past, present, future)	Acceptance Predictability Empathy	Recognise their history, hopes, transitions; support predictable pathways; show empathy for their trajectory	Focus only on ‘fixing now’; ignore their past or future; treat them as static or defined by a label
**Sense of place** (feeling at home in one's body, environment, surroundings)	Sensory Predictability	Adapt physical/sensory environment to help them feel safe and at home; support predictability of setting	Use chaotic or overstimulating settings; ignore sensory distress; expect adaptation without environmental change
**Embodiment** (holistic person‐in‐body, acknowledging physical, sensory, and emotional together)	Sensory Empathy Acceptance	Respect sensory regulation, body signals, fatigue; show empathy for embodied needs; accept person‐in‐body	Treat the person as only mind/behaviour; ignore sensory or physical cues; force activity, ignoring fatigue or sensory overload

## 5 Conclusion

All healthcare professionals, including radiographers, ought to use more inclusive, person‐centred language when communicating with and about neurodivergent individuals. We need cultural change to protect neurodivergent wellbeing, both for patients and practitioners in healthcare. To achieve this, we need to educate our workforce intentionally and with empathy. We need to raise awareness of neurodivergence and increase understanding of the impacts of the language we use on neurodivergent populations. This is a call for action for a systemic change in language use, communication and approach as an expression of humanised care. In recognising the power of words to respect or harm, radiographers can contribute to the wider change of narrative and practice, while culminating a shift from disorder to diversity, redefining what compassionate, person‐centred care truly means for autistic and neurodivergent patients.

## 6 Reflexivity and Positionality Statement

The authors of this study are diverse in gender (6 men, 9 women) and neurodiversity (6 = neurotypical, 9 = neurodivergent). The team also includes 12 people who identify as straight and 3 as queer, 1 black and 14 white scholars. Our researchers come from a range of clinical (*n* = 7), academic (*n* = 8), research backgrounds (*n* = 10), and third sector (*n* = 1). The first author is a registered PhD student within the CRRAG research group, working on increasing accessibility of autistic and neurodivergent people in healthcare. The team is committed to person‐centred, experience‐sensitive and neurodiversity‐friendly education, research and practice. All authors are strong advocates of the need for neurodiversity training and appreciate the importance of respectful language for accessibility and inclusion of neurodivergent people in healthcare and all aspects of life.

## Conflicts of Interest

The authors declare no conflicts of interest.

## Linked Articles

This article is linked to Wickramasinghe et al. papers. To view these article, visit https://onlinelibrary.wiley.com/doi/10.1002/jmrs.70023.

## Data Availability

Data sharing not applicable to this article as no datasets were generated or analysed during the current study.

## References

[1] T. Karaminis , M. Botha , S. Longley , et al., “Language Matters in British Newspapers: A Participatory Analysis of the Autism UK Press Corpus,” Autism in Adulthood 7, no. 6 (2024): 698–711, 10.1089/aut.2023.0105. PMC12851867 41625312

[2] K. Bottema‐Beutel , S. K. Kapp , J. N. Lester , N. J. Sasson , and B. N. Hand , “Avoiding Ableist Language: Suggestions for Autism Researchers,” Autism in Adulthood 3 (2021): 18–29, 10.1089/aut.2020.0014. 36601265 PMC8992888

[3] R. Wickramasinghe and G. McLean , “Improving Procedure Completion and Engagement of Neurodivergent Patients in Medical Imaging: A Systematic Review,” Journal of Medical Radiation Sciences (2025): 1–8, https://onlinelibrary.wiley.com/doi/10.1002/jmrs.70023. 10.1002/jmrs.70023 PMC13398792 40970542

[4] C. Beardmore , A. England , C. Cruwys , D. Carrié , and European Federation of Radiographer Societies, and the European Society of Radiology , “How Can Effective Communication Help Radiographers Meet the Expectations of Patients—COMMUNICATION—A Joint Statement by the ESR & EFRS,” Insights Into Imaging 15 (2024): 300, 10.1186/s13244-024-01868-5. 39699740 PMC11659540

[5] A. F. Reitan and A. Sanderud , “Communicating Radiation Risk to Patients: Experiences Among Radiographers in Norway,” Journal of Medical Imaging and Radiation Sciences 51 (2020): S84–S89, 10.1016/j.jmir.2020.06.011. 32741740

[6] N. Pollard , M. Lincoln , G. Nisbet , and M. Penman , “Patient Perceptions of Communication With Diagnostic Radiographers,” Radiography 25 (2019): 333–338, 10.1016/j.radi.2019.04.002. 31582241

[7] I. Manouilenko and S. Bejerot , “Sukhareva—Prior to Asperger and Kanner,” Nordic Journal of Psychiatry 69 (2015): 1761–1764, 10.3109/08039488.2015.1005022. 25826582

[8] C. Simmonds and G. E. Sukhareva , “The First Account of the Syndrome Asperger Described? Part 2: The Girls,” European Child & Adolescent Psychiatry 29 (2020): 549–564, 10.1007/s00787-019-01371-z. 31367779

[9] D. A. Sher and J. L. Gibson , “Pioneering, Prodigious and Perspicacious: Grunya Efimovna Sukhareva's Life and Contribution to Conceptualising Autism and Schizophrenia,” European Child & Adolescent Psychiatry 32 (2023): 475–490, 10.1007/s00787-021-01875-7. 34562153 PMC10038965

[10] L. Kanner , “Autistic Disturbances of Affective Contact,” Nervous Child 2 (1943): 217–250. 4880460

[11] H. Asperger , “Die “Autistischen Psychopathen” im Kindesalter,” Archiv f Psychiatrie 117 (1944): 76–136, 10.1007/BF01837709.

[12] S. Wolff , “The First Account of the Syndrome Asperger Described?: Translation of a Paper Entitled ?Die Schizoiden Psychopathien Im Kindesalter by Dr. G. E. Ssucharewa; Scientific Assistant, Which Appeared in 1926 in the Monatsschrift fur Psychiatrie Und Neurologie 60:235?261,” European Child & Adolescent Psychiatry 5 (1996): 119–132, 10.1007/BF00571671. 8908418

[13] B. Bettelheim , The Empty Fortress: Infantile Autism and the Birth of the Self Oxford, England (Free Press of Glencoe, 1967).

[14] S. Baron‐Cohen , Mindblindness: An Essay on Autism and Theory of Mind (MIT Press, 1995), 10.7551/mitpress/4635.001.0001.

[15] S. K. Kapp , K. Gillespie‐Lynch , L. E. Sherman , and T. Hutman , “Deficit, Difference, or Both? Autism and Neurodiversity,” Developmental Psychology 49 (2013): 59–71, 10.1037/a0028353. 22545843

[16] S. K. Kapp , ed., Autistic Community and the Neurodiversity Movement: Stories From the Frontline Singapore (Springer, 2020), 10.1007/978-981-13-8437-0.

[17] M. Botha , “Academic, Activist, or Advocate? Angry, Entangled, and Emerging: A Critical Reflection on Autism Knowledge Production,” Frontiers in Psychology 12 (2021): 727542, 10.3389/fpsyg.2021.727542. 34650484 PMC8506216

[18] N. Keates , F. Martin , and K. E. Waldock , “Autistic People's Perspectives on Functioning Labels and Associated Reasons, and Community Connectedness,” Journal of Autism and Developmental Disorders 55 (2025): 1318–1328, 10.1007/s10803-024-06316-3. 38507152

[19] D. Kanagasingam , L. Hurd , and M. Norman , “Integrating Person‐Centred Care and Social Justice: A Model for Practice With Larger‐Bodied Patients,” Medical Humanities 49 (2023): 436–446, 10.1136/medhum-2021-012351. 36635073

[20] B. Bogaert , “Need for Patient‐Developed Concepts of Empowerment to Rectify Epistemic Injustice and Advance Person‐Centred Care,” Journal of Medical Ethics 47 (2021): e15, 10.1136/medethics-2020-106558. 33246999

[21] M. Botha , B. Dibb , and D. M. Frost , ““Autism Is Me”: An Investigation of How Autistic Individuals Make Sense of Autism and Stigma,” Disability & Society 37 (2022): 427–453, 10.1080/09687599.2020.1822782.

[22] S. M. Bury , R. Jellett , A. Haschek , M. Wenzel , D. Hedley , and J. R. Spoor , “Understanding Language Preference: Autism Knowledge, Experience of Stigma and Autism Identity,” Autism 27 (2023): 1588–1600, 10.1177/13623613221142383. 36510834

[23] M. Doherty , S. Neilson , J. O'Sullivan , et al., “Barriers to Healthcare and Self‐Reported Adverse Outcomes for Autistic Adults: A Cross‐Sectional Study,” BMJ Open 12 (2022): e056904, 10.1136/bmjopen-2021-056904. PMC8883251 35193921

[24] Department of Work and P ensions , The Buckland Review of Autism Employment: Report and Recommendations, 2024 accessed at, January 26th, 2026, https://www.gov.uk/government/publications/the‐buckland‐review‐of‐autism‐employment‐report‐and‐recommendations/the‐buckland‐review‐of‐autism‐employment‐report‐and‐recommendations.

[25] M. Doherty , S. McCowan , and S. C. Shaw , “Autistic SPACE: A Novel Framework for Meeting the Needs of Autistic People in Healthcare Settings,” British Journal of Hospital Medicine 84 (2023): 1–9, 10.12968/hmed.2023.0006. 37127416

[26] J. L. L. Csecs , V. Iodice , C. L. Rae , et al., “Joint Hypermobility Links Neurodivergence to Dysautonomia and Pain,” Frontiers in Psychiatry 2, no. 12 (2022): 786916, 10.3389/fpsyt.2021.786916. PMC8847158 35185636

[27] B. Donaghy , D. Moore , and J. Green , “Co‐Occurring Physical Health Challenges in Neurodivergent Children and Young People: A Topical Review and Recommendation,” Child Care in Practice 29, no. 1 (2023): 3–21, 10.1080/13575279.2022.2149471.

[28] E. O'Nions , C. El Baou , A. John , et al., “Life Expectancy and Years of Life Lost for Adults With Diagnosed ADHD in the UK: Matched Cohort Study,” British Journal of Psychiatry 226, no. 5 (2025): 1–8, 10.1192/bjp.2024.199. PMC7617439 39844532

[29] E. Cage , J. Di Monaco , and V. Newell , “Understanding, Attitudes and Dehumanisation Towards Autistic People,” Autism 23 (2019): 1373–1383, 10.1177/1362361318811290. 30463431

[30] A. Grant , S. Turner , S. C. K. Shaw , et al., ““I Am Afraid of Being Treated Badly if I Show It”: A Cross‐Sectional Study of Healthcare Accessibility and Autism Health Passports Among UK Autistic Adults,” PLoS One 19 (2024): e0303873, 10.1371/journal.pone.0303873. 38809913 PMC11135756

[31] S. K. Au‐Yeung , M. Freeth , and A. R. Thompson , “‘Am I Gonna Regret This?’: The Experiences of Diagnostic Disclosure in Autistic Adults,” Autism 29, no. 8 (2025): 2181–2192, 13623613251337504, 10.1177/13623613251337504. 40357870 PMC12255832

[32] D. Miller , J. Rees , and A. Pearson , ““Masking Is Life”: Experiences of Masking in Autistic and Nonautistic Adults,” Autism in Adulthood 3, no. 4 (2021): 330–338, 10.1089/aut.2020.0083. 36601640 PMC8992921

[33] E. Cage and Z. Troxell‐Whitman , “Understanding the Reasons, Contexts and Costs of Camouflaging for Autistic Adults,” Journal of Autism and Developmental Disorders 49 (2019): 1899–1911, 10.1007/s10803-018-03878-x. 30627892 PMC6483965

[34] S. C. Shaw , L. Carravallah , M. Johnson , et al., “Barriers to Healthcare and a ‘Triple Empathy Problem’ May Lead to Adverse Outcomes for Autistic Adults: A Qualitative Study,” Autism 28, no. 7 (2023): 1746–1757, 13623613231205629, 10.1177/13623613231205629. 37846479 PMC11191657

[35] LeDeR report , Eighth ‘Learning From Lives and Deaths – People With a Learning Disability and Autistic People’ (LeDeR), 2026 accessed at, February 5th, 2026, https://questions‐statements.parliament.uk/written‐statements/detail/2026‐01‐27/hcws1276.

[36] L. Kenny , C. Hattersley , B. Molins , C. Buckley , C. Povey , and E. Pellicano , “Which Terms Should Be Used to Describe Autism? Perspectives From the UK Autism Community,” Autism 20 (2016): 442–462, 10.1177/1362361315588200. 26134030

[37] S. M. Bury , R. Jellett , J. R. Spoor , and D. Hedley , ““It Defines Who I am” or “It's Something I Have”: What Language Do [Autistic] Australian Adults [on the Autism Spectrum] Prefer?,” Journal of Autism and Developmental Disorders 53 (2023): 677–687, 10.1007/s10803-020-04425-3. 32112234

[38] S.‐M. Fecteau , C. L. Normand , G. Normandeau , et al., ““Not a Trouble”: A Mixed‐Method Study of Autism‐Related Language Preferences by French‐Canadian Adults From the Autism Community,” Neurodiversity 2 (2024): 27546330241253696, 10.1177/27546330241253696.

[39] B. Potts , N. Smith , and C. Malamateniou , “Respectful Language in Autism Research: In Response to Abdelrahman Et al. ‘Exploration of Radiographers’ Knowledge, Attitudes, and Practices in Delivering Healthcare to Children With Autism Spectrum Disorder,” Radiography 30 (2024): 702–703, 10.1016/j.radi.2024.02.003. 38412769

[40] R. Monk , A. J. O. Whitehouse , and H. Waddington , “The Use of Language in Autism Research,” Trends in Neurosciences 45 (2022): 791–793, 10.1016/j.tins.2022.08.009. 36184384

[41] P. Bradshaw , C. Pickett , M. L. Van Driel , K. Brooker , and A. Urbanowicz , “‘Autistic’ or ‘With Autism’? Why the Way General Practitioners View and Talk About Autism Matters,” Australian Journal of General Practice 50 (2021): 104–108, 10.31128/AJGP-11-20-5721. 33634274

[42] H. M. Natri , O. Abubakare , K. Asasumasu , et al., “Anti‐Ableist Language Is Fully Compatible With High‐Quality Autism Research: Response to Singer Et al. (2023),” Autism Research 16 (2023): 673–676, 10.1002/aur.2928. 37087601

[43] J. M. Harvey‐Lloyd , A. Clements , N. Sims , and A. E. Harvey‐Lloyd , “Exploring the Experiences of Parents of Autistic Children When Attending the Diagnostic Imaging Department for an X‐Ray Examination,” Radiography 30 (2024): 28–36, 10.1016/j.radi.2023.09.014. 37866155

[44] C. Nicolaidis , D. M. Raymaker , E. Ashkenazy , et al., ““Respect the Way I Need to Communicate With You”: Healthcare Experiences of Adults on the Autism Spectrum,” Autism 19 (2015): 824–831, 10.1177/1362361315576221. 25882392 PMC4841263

[45] The Double Empathy Problem n.d. accessed at, November 1, 2025, https://www.autism.org.uk/advice‐and‐guidance/professional‐practice/double‐empathy.

[46] D. E. M. Milton , “On the Ontological Status of Autism: The ‘Double Empathy Problem’,” Disability & Society 27 (2012): 883–887, 10.1080/09687599.2012.710008.

[47] Community Against Prejudice Towards Autistic People (CAPTAP) , How to Talk About Autistic Ways of Being. CAPTAP, 2023 accessed at, November 1, 2025, https://captapnetwork.wordpress.com/2023/09/19/how‐to‐talk/.

[48] R. T. Cheang , M. Skjevling , A. I. Blakemore , V. Kumari , and I. Puzzo , “Do You Feel Me? Autism, Empathic Accuracy and the Double Empathy Problem,” Autism 29, no. 9 (2024): 2315–2327, 13623613241252320, 10.1177/13623613241252320. 38757626 PMC12332230

[49] D. Murray , M. Lesser , and W. Lawson , “Attention, Monotropism and the Diagnostic Criteria for Autism,” Autism 9 (2005): 139–156, 10.1177/1362361305051398. 15857859

[50] P. L. Howard and F. Sedgewick , “‘Anything but the Phone!’: Communication Mode Preferences in the Autism Community,” Autism 25 (2021): 2265–2278, 10.1177/13623613211014995. 34169750

[51] S. R. Arnold , G. Bruce , J. Weise , C. J. Mills , J. N. Trollor , and K. Coxon , “Barriers to Healthcare for Australian Autistic Adults,” Autism 28 (2024): 301–315, 10.1177/13623613231168444. 37161777 PMC10851652

[52] L. J. Camilleri , K. Maras , and M. Brosnan , “Supporting Autistic Communities Through Parent‐Led and Child/Young Person‐Led Digital Social Story Interventions: An Exploratory Study,” Frontiers in Digital Health 6 (2024): 1355795, 10.3389/fdgth.2024.1355795. 38545445 PMC10965630

[53] NHS England , Making Information and the Words We Use Accessible accessed at, November 1, 2025, https://www.england.nhs.uk/learning‐disabilities/about/get‐involved/involving‐people/making‐information‐and‐the‐words‐we‐use‐accessible/.

[54] N. Stogiannos , J. M. Harvey‐Lloyd , A. Brammer , et al., “Toward Autism‐Friendly Magnetic Resonance Imaging: Exploring Autistic Individuals' Experiences of Magnetic Resonance Imaging Scans in the United Kingdom, a Cross‐Sectional Survey,” Autism in Adulthood 5 (2023): 248–262, 10.1089/aut.2022.0051. 37663444 PMC10468562

[55] C. T. Keating , L. Hickman , J. Leung , et al., “Autism‐Related Language Preferences of English‐Speaking Individuals Across the Globe: A Mixed Methods Investigation,” Autism Research 16 (2023): 406–428, 10.1002/aur.2864. 36474364 PMC10946540

[56] A. Taboas , K. Doepke , and C. Zimmerman , “Preferences for Identity‐First Versus Person‐First Language in a US Sample of Autism Stakeholders,” Autism 27 (2023): 565–570, 10.1177/13623613221130845. 36237135

[57] G. L. Williams , R. Ellis , H. Axbey , et al., “Autistic, Hysteric: Inequity in Health Care for Autistic People Assigned Female at Birth in the United Kingdom,” in The Double Empathy Reader: Exploring Theory, Neurodivergent Lived Experience and Implications for Practice Shoreham‐by‐Sea (UK), edited by D. Milton (Pavilion Publishing and Media Ltd, 2025), https://www.ncbi.nlm.nih.gov/books/NBK615817/. 40638767

[58] E. McGreevy , A. Quinn , R. Law , et al., “An Experience Sensitive Approach to Care With and for Autistic Children and Young People in Clinical Services,” Journal of Humanistic Psychology 66, no. 1 (2024): 107–133, 10.1177/00221678241232442.

